# Supplementary material for: Degradation of the Tumor Suppressor PDCD4 Is Impaired by the Suppression of p62/SQSTM1 and Autophagy
Source: Cells. 2020 Jan 15;9(1):218. doi: 10.3390/cells9010218 (PMC7016974; doi:10.3390/cells9010218)
Supplement: Supplementary file 1 [file cells-09-00218-s001.zip › cells-663520-supplementary.docx]

**Supplementary Figures**


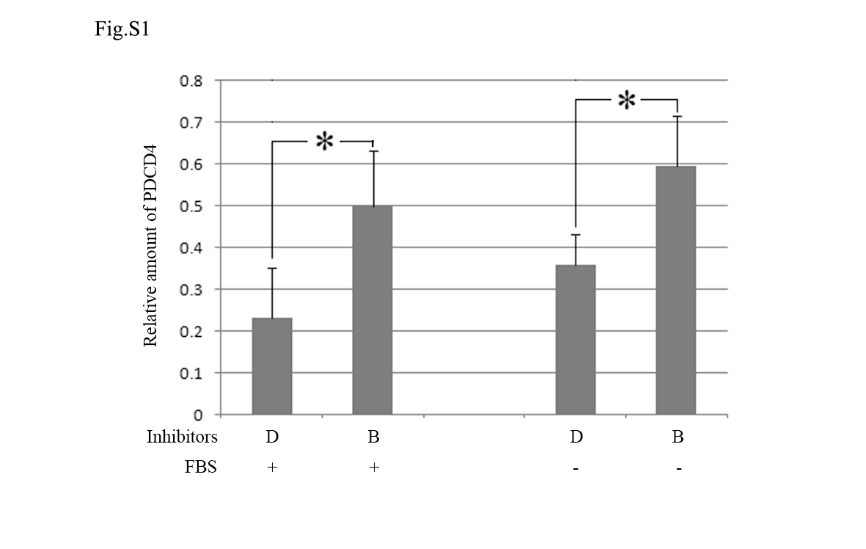


**Fig. S1.** The autophagy inhibitor bafilomycin A1 upregulated PDCD4 levels in Huh7 cells. Huh7 cells are treated with 10 µM bafilomycin A1 (B) or DMSO (D) as a control in the presence or absence of FBS. At four hours after the addition of the inhibitors, cells were subjected to Western blotting. The PDCD4 protein brands were determined by image J and normalized to β-actin. Data were an average of three independent experiments. Statistically significant differences (P<0.05) are represented by asterisk (*) mark.


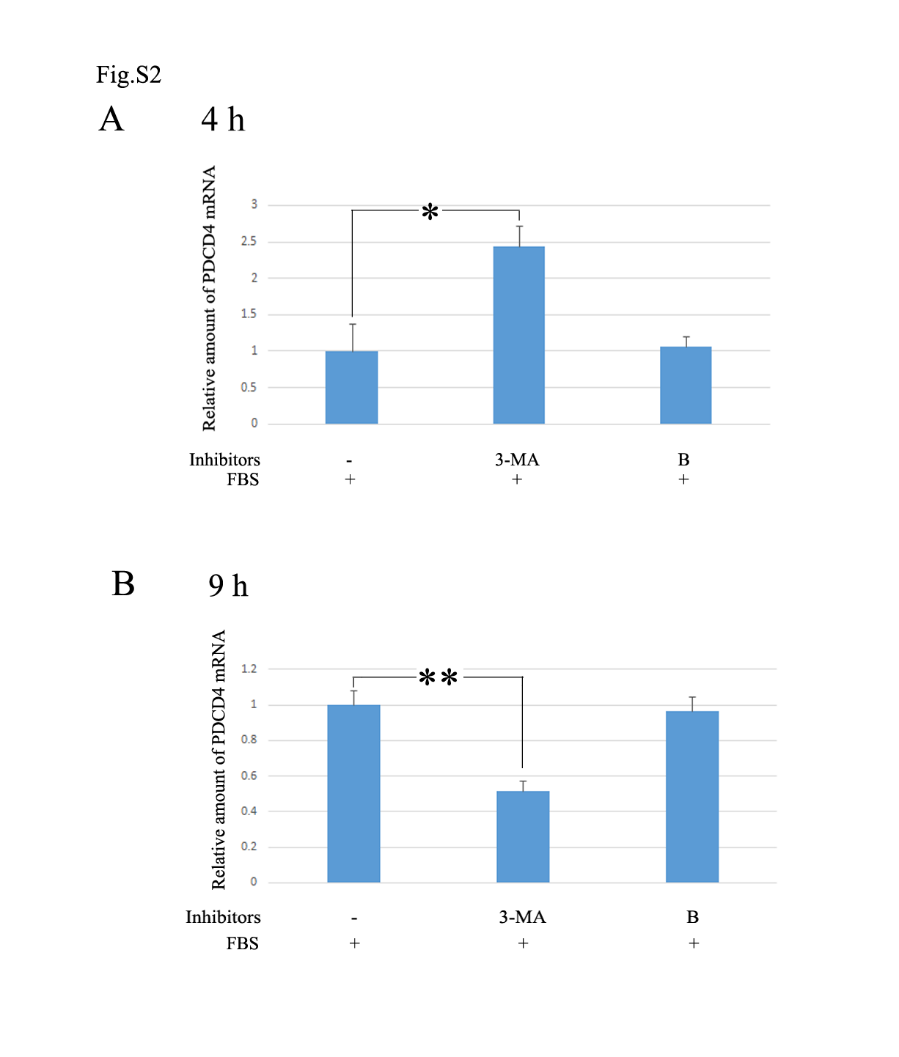


**Fig. S2.** The PDCD4 mRNA levels were not changed by bafilomycinA1 but modulated by 3-MA. 5x10^5^ Huh7 cells were cultured in 60-mm culture dishes in the presence of DMEM+10% FBS until 80-90 % confluency. The old medium was replaced with DMEM+10% FBS. Before adding new medium, the dishes were thoroughly washed twice with DMEM. The cells were treated with 10 µM bafilomycin A1 (B) and 5 mM 3-MA (3-MA). After 4 and 9 hours of treatment the cells were collected for RNA isolation. Data were an average of three independent experiments. Statistically significant differences (P<0.05 and P<0.005) are represented by asterisk (*) and (**) marks respectively.


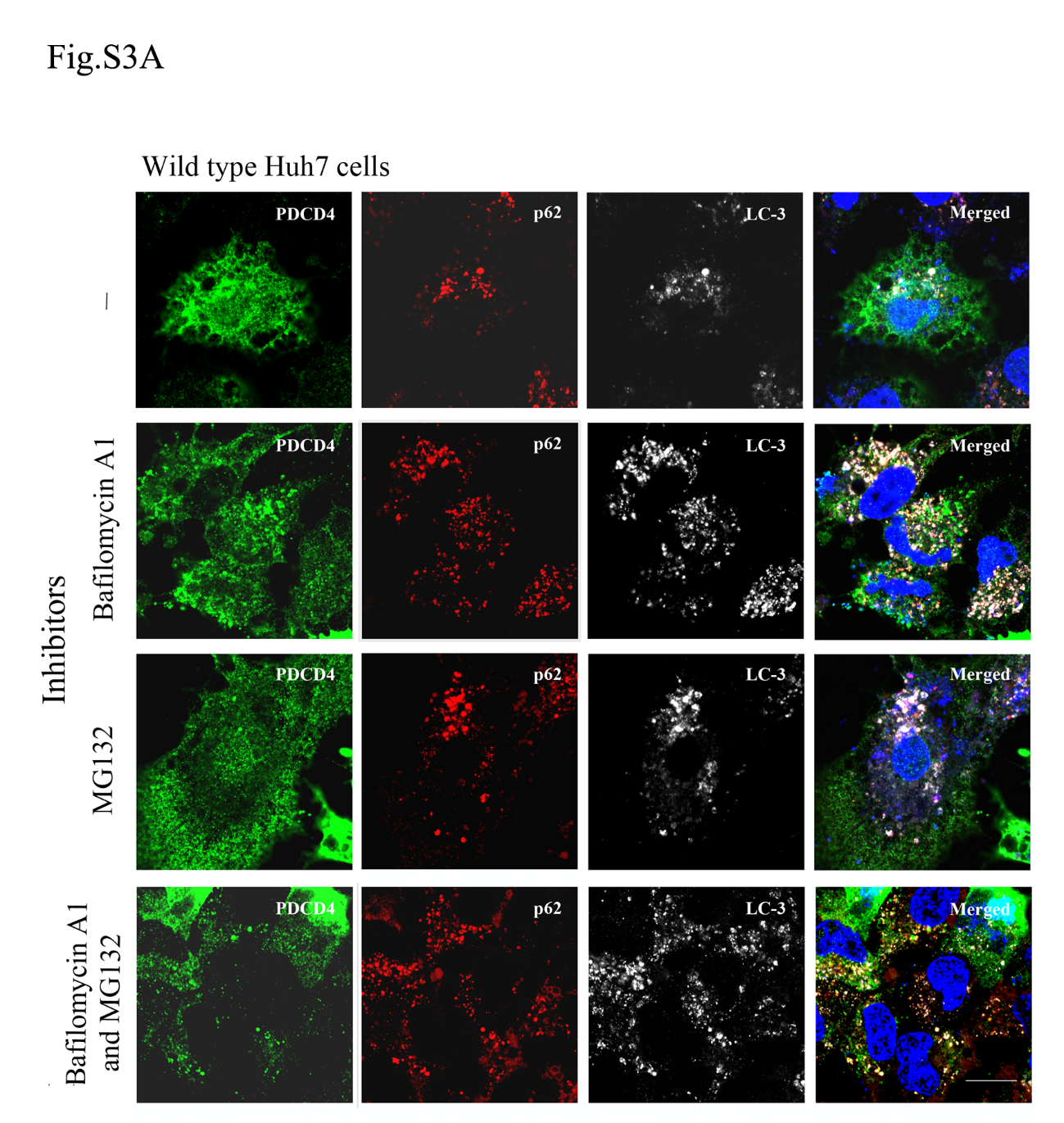


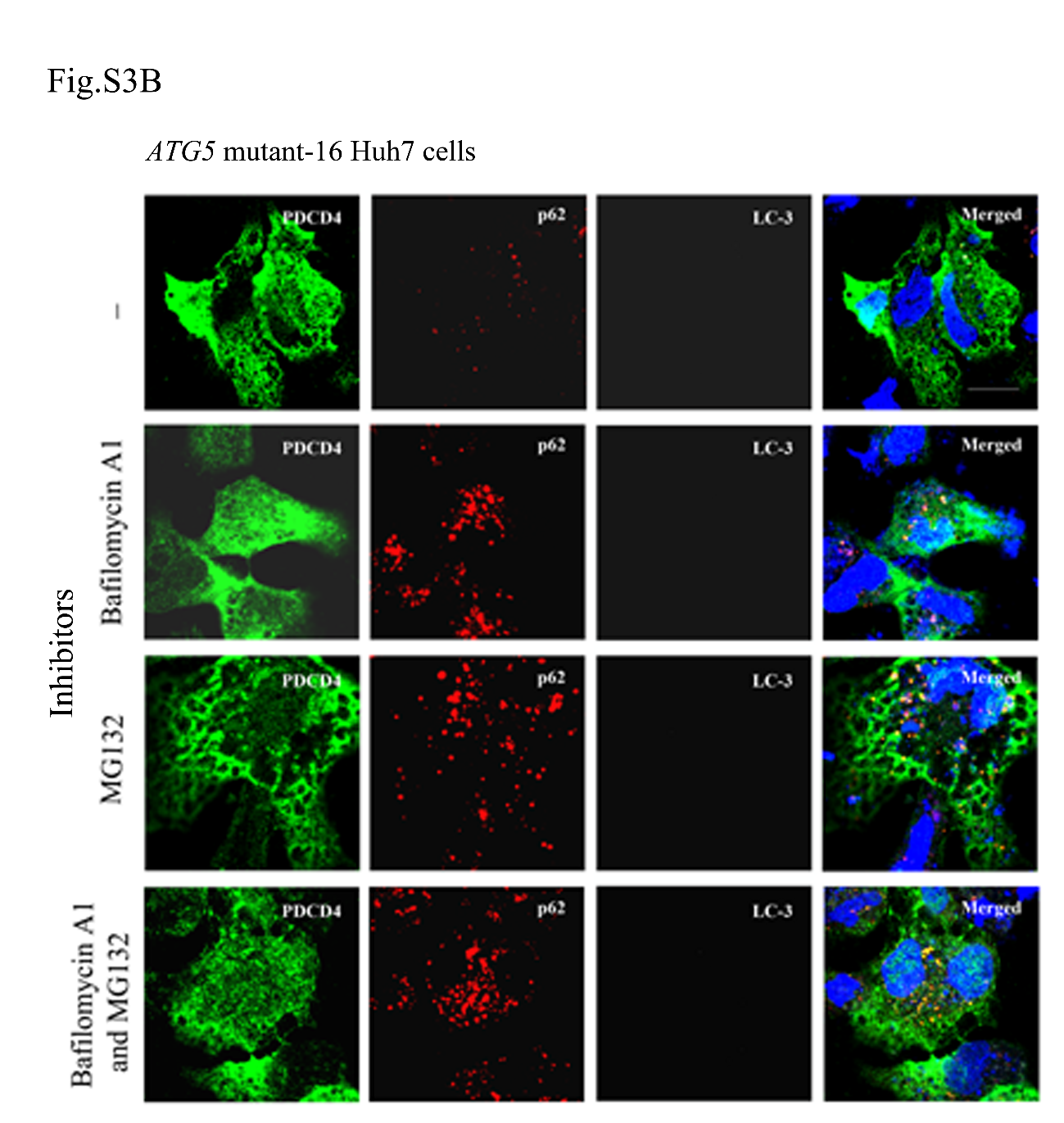


**Fig. S3.** Colocalization of PDCD4, p62 and LC3 was increased in the presence of both bafilomycin A1 and/or MG132 in both wild-type (A) and *ATG5*-16 mutant (B) Huh7 cells. (A) A total of 1.5×10^5^ Huh7 cells were seeded onto a cover glass in 35-mm dishes and cultured until 70%-80% confluency. Cells were then transfected with PDCD4 plasmid and cultured for a further 20-22 hours in the presence of DMEM+10% FBS (without antibiotics). The old medium was replaced with DMEM. However, before adding new medium the dishes were washed twice with DMEM. Culture was continued for 4 h in the absence of inhibitor or in the presence of bafilomycin A1 and/or MG132, as indicated in the figure. The cells were fixed with 4% paraformaldehyde. The detailed procedure was described in the Methods section. Images were captured using an LSM-880 confocal microscope. (B) This figure showed the colocalization of PDCD4, p62 and LC3 in the *ATG5* mutant-16 Huh7 cells line. The same protocol for (A) was used for this experiment. Although the treatments failed to stimulate the LC3 expression or formation of particles, the colocalization of PDCD4 and p62 was stimulated. Bar=300 µm in both figures.
